# Methodological advances of bioanalysis and biochemical targeting of intracellular G‐quadruplexes

**DOI:** 10.1002/EXP.20210214

**Published:** 2022-02-27

**Authors:** Xucong Teng, Yicong Dai, Jinghong Li

**Affiliations:** ^1^ Department of Chemistry, Center for BioAnalytical Chemistry, Key Laboratory of Bioorganic Phosphorus Chemistry & Chemical Biology Tsinghua University Beijing China

**Keywords:** biochemical targeting, cell imaging, G‐quadruplex, high‐throughput sequencing, intracellular detection, proteomics

## Abstract

G‐quadruplexes (G4s) are a kind of non‐canonical nucleic acid secondary structures, which involve in various biological processes in living cells. The relationships between G4s and human diseases, such as tumors, neurodegenerative diseases, and viral infections, have attracted great attention in the last decade. G4s are considered as a promising new target for disease treatment. For instance, G4 ligands are reported to be potentially effective in SARS‐COV‐2 treatment. However, because of the lack of analytical methods with high performance for the identification of intracellular G4s, the detailed mechanisms of the biofunctions of G4s remain elusive. Meanwhile, through demonstrating the principles of how the G4s systematically modulate the cellular processes with advanced detection methods, biochemical targeting of G4s in living cells can be realized by chemical and biological tools and becomes useful in biomedicine. This review highlights recent methodological advances about intracellular G4s and provides an outlook on the improvement of the bioanalysis and biochemical targeting tools of G4s.

## Graphical abstract

G‐quadruplexes involve in various biological processes in living cells, and are considered as a promising new target for disease therapeutics, but the detailed mechanism of G4 biofunction remains elusive. This review highlights recent methodological advances about intracellular G‐quadruplexes and provides an outlook on the improvement of the bioanalysis and biochemical targeting tools of G‐quadruplexes.

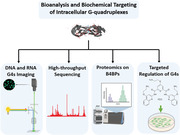

## 1 INTRODUCTION

**FIGURE 1:**
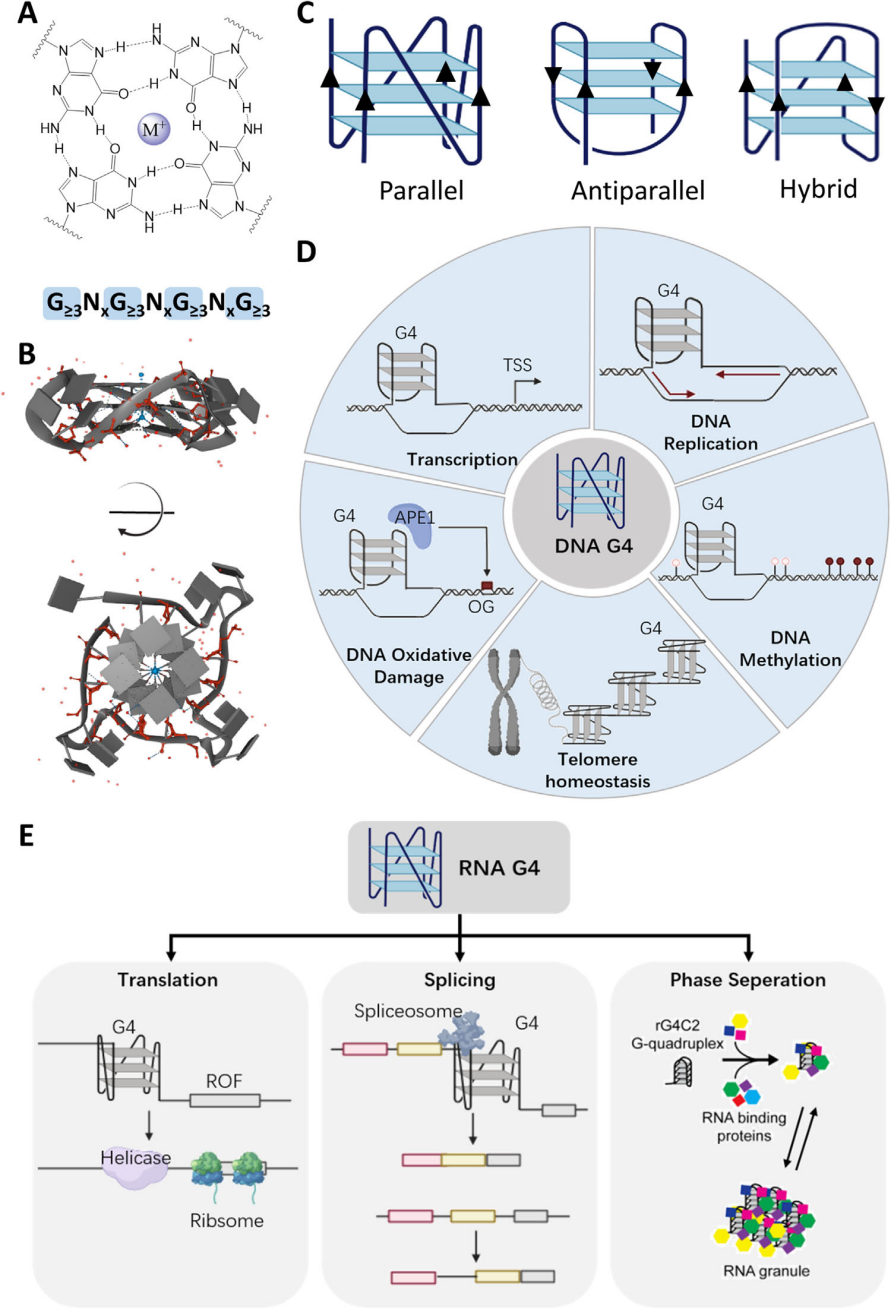
G‐quadruplex structure and biology. (A) The chemical structure of a G‐quartet and the consensus sequence formula of G4s. *x* represents the number of nucleotides in the loops. Monovalent ion sits within the G‐quartet for stabilization. (B) The X‐ray crystal structure of a parallel G4 (PDB: 1KF1).^[^ ^19^ ^]^ (C) Representative topologies of G4 structures. (D) Representative DNA‐G4‐associated biological processes. (E) Representative RNA‐G4‐associated biological processes. Phase seperation panel: Adapted with permission.^[^ ^59^ ^]^ Copyright 2017, Elsevier. TSS, transcription start site; OG, 8‐oxoGuanine; ORF, open reading frame

G‐quadruplexes (G4s) are a class of non‐canonical nucleic acid secondary structures. Four guanine bases self‐associate via Hoogsteen hydrogen bonds into a square planar conformation, namely, G‐quartet (Figure 1A). Two or three layers of G‐quartets stack into peculiar structural motifs, which are called G4s (Figure 1B). Monovalent ions bind to the G‐quartets via coordinating with the C6 carbonyl oxygen of the guanine bases, which can stabilize G4 structures (Stabilization effect: K^+ ^> Na^+ ^> > Li^+^).^[^ ^1^, ^2^ ^]^ G4s undergo the formula G_≥3_N*_x_* G_≥3_N*_x_* G_≥3_N*_x_* G_≥3_ (*x* = 1–7), while there are also other forms of G4s, such as two‐quartet, long‐loop, and bulge G4s.^[^ ^3^, ^4^ ^]^ The sequences between G‐tracts form three loop regions. The sequence component and length of the loops play an important role in the stability of G4 structures.^[^ ^2^, ^5^ ^]^ In general, G4s are more stable than other competing nucleic acid structures.^[^ ^6^ ^]^ G4s can form in both DNA and RNA, and have diverse substructures.^[^ ^7^ ^]^ Dictated by the orientation of G‐tracts, DNA G4s can fold into parallel, anti‐parallel, and mixed topologies (Figure 1C), while RNA G4s have only the parallel topology.

G4s are widely present in the genome^[^ ^8^ ^]^ and transcriptome^[^ ^9^ ^]^ of living cells. There are more than one million G4‐forming sequences in human and mouse cells. G4s participate in various cellular processes related to gene expression, such as transcription,^[^ ^10^ ^]^ translation,^[^ ^11^ ^]^ DNA replication,^[^ ^12^ ^]^ and so on. In addition, G4s have been reported to have critical regulatory functions in human diseases, including tumors,^[^ ^13^ ^]^ neurodegenerative diseases,^[^ ^14^ ^]^ and viral infections.^[^ ^15^ ^]^ In this way, G4s have been regarded as a potential therapeutic target.^[^ ^8^, ^16^, ^17^ ^]^ An important example of G4‐based therapeutics is that targeting the G4 in the SARS‐COV‐2 genomic RNA with a small molecular ligand is proved to be a potential treatment for COVID‐19.^[^ ^18^ ^]^ Therefore, there has been a recent surge in the study towards understanding G4s in living cells and organisms for the development of biomedicine applications based on intracellular G4s. However, limited by analytical methods, the detailed mechanisms of G4‐dependent biofunctions remain unclear.^[^ ^3^, ^9^ ^]^ For instance, it is hard to specifically detect G4s in an individual gene, while it is necessary to characterize the G4 in every single gene, to clarify its detailed molecular mechanism in a certain gene pathway.

The prerequisite for revealing the biofunction of G4s and developing novel G4 mediated biological applications is to establish a powerful toolkit of detection and characterization methods. In the last decade, various biophysical methods for characterizing G4‐forming oligonucleotides in vitro have been established, including circular dichroism (CD),^[^ ^20^ ^]^ fluorescence resonance energy transfer,^[^ ^21^ ^]^ ultraviolet‐visible spectrophotometry,^[^ ^22^ ^]^ nuclear magnetic resonance,^[^ ^23^ ^]^ surface‐enhancedRaman scattering,^[^ ^24^ ^]^ and so on. These methods have greatly expanded our understanding of G4 structures and their biochemical properties. However, in vitro experiments cannot reflect the real properties of G4s in the cells. Therefore, it is necessary to study the function and regulation of intracellular G4s in situ. Identification and characterization of G4s in cellular context raise higher requirements on analytical methods. Firstly, the in situ intracellular physiological state needs to be maintained to the greatest extent.^[^ ^8^ ^]^ Secondly, the G4s in the cells may interact with other nucleic acids and proteins, resulting in a very complicated chemical environment, thus the probes of G4s are susceptible to be interfered from non‐specific binding. Thirdly, there are millions of G4s in the cells, which are very difficult to be differentiated.^[^ ^25^ ^]^ In the past ten years, methodological advances such as cell imaging, high‐throughput sequencing, and proteomic technologies have been developed to systematically identify and characterize G4s in the cells. Although there are other methods that can be useful in intracellular G4s, these three types of methods have the greatest power to advance our understanding of G4 biology. Moreover, based on the knowledge of G4 biology, several chemical and biological tools have been established to regulate the biofunction of G4s in vivo,^[^ ^3^, ^26^ ^]^ which lay the foundation for G4‐based biomedical applications.

As early as 10 years ago, G4s were considered as targets for therapeutics. In the past decade, some progress has been made in the development and testing of G4‐based therapeutic methods, such as G4 ligands for anti‐tumor treatment and the detailed mechanism underlying G4‐related tumors. However, G4‐based biomedical applications are still in their infancy. Methodological advances are under demand to promote this field. On the one hand, improved bioanalytical methods can drive the discovery of the overall mechanism of G4‐mediated intracellular processes. On the other hand, it is essential to expand the biochemical toolkits for targeting G4. Herein, we believe that it is necessary to illustrate the currently applicable methods and highlight the recent advances about intracellular G4s. Importantly, discussing their principles, application scenarios, advantages and disadvantages can provide a guidance of the future improvement of intracellular G4 bioanalysis and biochemical targeting. In this review, we first briefly introduce the biological functions of G4s. Then, we overview G4 probes as the key molecular tools required for G4 detection and biochemical targeting, and focus on the screening methods of these probes. Next, the bioanalytical technologies of intracellular G4s are discussed from three categories: imaging, high‐throughput sequencing, and proteomic methods. Meanwhile, the biochemical targeting tools of G4s in living cells are summarized. Finally, we point out the currently unresolved questions in G4 bioanalysis and biochemical targeting.

## 2 MAJOR BIOFUNCTIONS OF G4

G4s are abundantly present in the genome and transcriptome, and the ever‐increasing number of discoveries has revealed that G4s participate in various biological processes related to DNA and RNA. G4s function in two major ways. Firstly, due to its high stability, DNA polymerase, RNA polymerase, and ribosome can be stalled by downstream G4s.^[^ ^27^ ^]^ Secondly, many endogenous proteins have been proved to bind with DNA and/or RNA G4s, in other words, G4s function as an assembly element to recruit additional regulatory proteins.^[^ ^9^ ^]^ The complex and diverse biofunctions of G4s have already been reviewed elsewhere.^[^ ^8^, ^26^, ^28^ ^]^ However, what G4s do in the cells remains a mainly unanswered question. Herein, we briefly introduce the major biofunction of G4s to facilitate the understanding of the scientific meaning of G4 bioanalysis and biochemical targeting.

### 2.1 Biofunctions of DNA G4s

Transcriptional regulation through G4s has been demonstrated by many studies,^[^ ^29^, ^30^, ^31^ ^]^ which is the most important biofunction of DNA G4s (Figure 1D). Computational identification of G4s using the consensus sequence resulted that there are more than 300,000 potential G4‐forming sequences in the human genome.^[^ ^32^, ^33^ ^]^ Moreover, G4‐forming sequences are significantly enriched in the promoter regions of genes,^[^ ^33^ ^]^ compared with the other regions of the genome. Therefore, G4s have the potential to involve in transcription. G4s in the promoter of human MYC gene have been detected in the cells, which proved that G4s in the promoters can fold in vivo.^[^ ^29^ ^]^ Through G4 ChIP‐seq, the formation of G4s in the chromatins has been further confirmed.^[^ ^34^ ^]^ These G4 sites are enriched in regulatory chromatins near the promoters of high abundance genes. In addition, many transcription‐related proteins can bind to G4 structures. For example, the binding sites of transcription‐associated helicases, XPB and XPD in the genome, significantly overlap with G4 motifs.^[^ ^31^ ^]^ Recently, Spiegel *et al*. further confirmed that G4s function as general binding hubs of various transcription factors to upregulate transcription.^[^ ^10^ ^]^ This finding was corroborated by related observations about the relationship between promoter G4s and cell‐specific transcriptome.^[^ ^35^ ^]^

G4s are involved in a wide range of DNA‐related processes. DNA replication is a finely regulated cellular process, in which DNA G4s play an important role. The Rif1 protein, which regulates DNA replication and DNA damage repair, can bind to the G4 structures to regulate the process of DNA replication.^[^ ^12^ ^]^ After being stabilized by small molecule ligands, G4s have been found to inhibit DNA replication,^[^ ^36^ ^]^ leading to histone demethylation and DNA methylation‐mediated gene silencing. Interestingly, G4s can also inhibit DNA methylation.^[^ ^37^ ^]^ DNMT1, a DNA methylase, binds to the G4 structure and its enzymatic activity is inhibited by G4s, resulting in G4‐dependent hypomethylation regions in the genome DNA.^[^ ^38^ ^]^

In addition, the relationship between G4 and telomere homeostasis has attracted much attention.^[^ ^39^ ^]^ The telomere repeat sequence TTAGGG matches to the consensus sequence of G4s, and many studies have shown that tandem G4s exist in the telomere region.^[^ ^40^ ^]^ Telomere binding protein, TREF2, not only binds to telomere G4s, but also binds to G4s in the other regions of chromatins.^[^ ^41^ ^]^

Finally, G4s are also related to DNA oxidative damage. The last G of the NGG sequence has the lowest redox potential,^[^ ^42^ ^]^ which exists in G4‐forming sequences. G4 sites are more susceptive to be oxidized by reactive oxygen species (ROS) to form 8‐oxo‐2′‐deoxyguanosine (8‐oxo‐dG).^[^ ^43^ ^]^ Some proteins related to DNA damage repair and transcription, such as APE1 and OGG1/2, can be recruited to the oxidation sites. In this way, promoter G4s are related to transcriptional activation in response to oxidative stress.^[^ ^44^ ^]^

### 2.2 Biofunctions of RNA G4s

G4s exist in many kinds of RNAs in the cellular transcriptome, including messenger RNAs (mRNAs), long noncoding RNAs, mitochondrial RNAs, microRNAs, telomere‐associated RNAs. Increasing evidence shows that G4s are widely involved in various RNA bioprocesses, which further confirms the complexity of G4 biology. In cellular mRNA, G4s are present in 5′‐UTR (untranslated region), 3′UTR, and CDS (coding sequence) regions. Similar to other stable secondary structures, G4s in mRNAs inhibit translation^[^ ^45^ ^]^ (Figure 1E). G4s in the 5′ UTR of mRNA have been clarified to inhibit translation in vitro.^[^ ^46^ ^]^ This effect can also be observed in living cells in many genes that contain G4s in 5′‐UTR.^[^ ^47^ ^]^ mRNA G4s hinder the formation of pre‐initiation complex by Steric constraints, which is the major principle of translation inhibition.^[^ ^17^ ^]^ Therefore, the effect of G4‐mediated translation inhibition is positively related to the stability of G4 structures.^[^ ^48^ ^]^ On the other hand, unfolding the G4 structures by RNA helicases can relieve the inhibition effect of G4s. For instance, translation of G4‐containing mRNAs is promoted by RNA helicases DHX9 and DHX36,^[^ ^11^, ^49^, ^50^ ^]^ and RNA G4s can also contribute to eIF4A‐mediated translation of proto‐oncogenes.^[^ ^51^ ^]^

In mRNAs, some G4s are located around the splicing sites, which have been found to regulate alternative splicing.^[^ ^52^ ^]^ Since the stable G4 structures mediate the recruitment of splicing‐related proteins, the abundance of different splicing variants can be altered by the folding and unfolding of G4s.^[^ ^53^, ^54^ ^]^ The regulation results of G4s around the splicing sites can be either increasing or inhibiting. The G4 in TERT mRNA intron 6 has been proved to inhibit splicing.^[^ ^55^ ^]^ On the contrary, the G4 in P53 mRNA intron 3 is essential for the successful splicing.^[^ ^56^ ^]^ However, the detailed mechanism of G4‐mediated splicing is unclear. Further data are under demand to fully elucidate the mechanism of cause and effect in these studies.

Phase separation is a newly discovered behavior of intracellular biomacromolecules, which provides a special way to gather specific molecules in the cells.^[^ ^57^ ^]^ RNA G4‐binding proteins mostly have intrinsically disordered arginine‐/glycine‐rich regions,^[^ ^58^ ^]^ which is important in phase separation process. Recently, it has been reported that RNA G4 can induce phase separation by recruiting proteins and promote the formation of RNA granules.^[^ ^59^ ^]^ These findings suggested a link between RNA G4 and phase separation, but the understanding of the molecular principle is limited.

## 3 G4 PROBES

### 3.1 G4 ligands and antibodies

**FIGURE 2:**
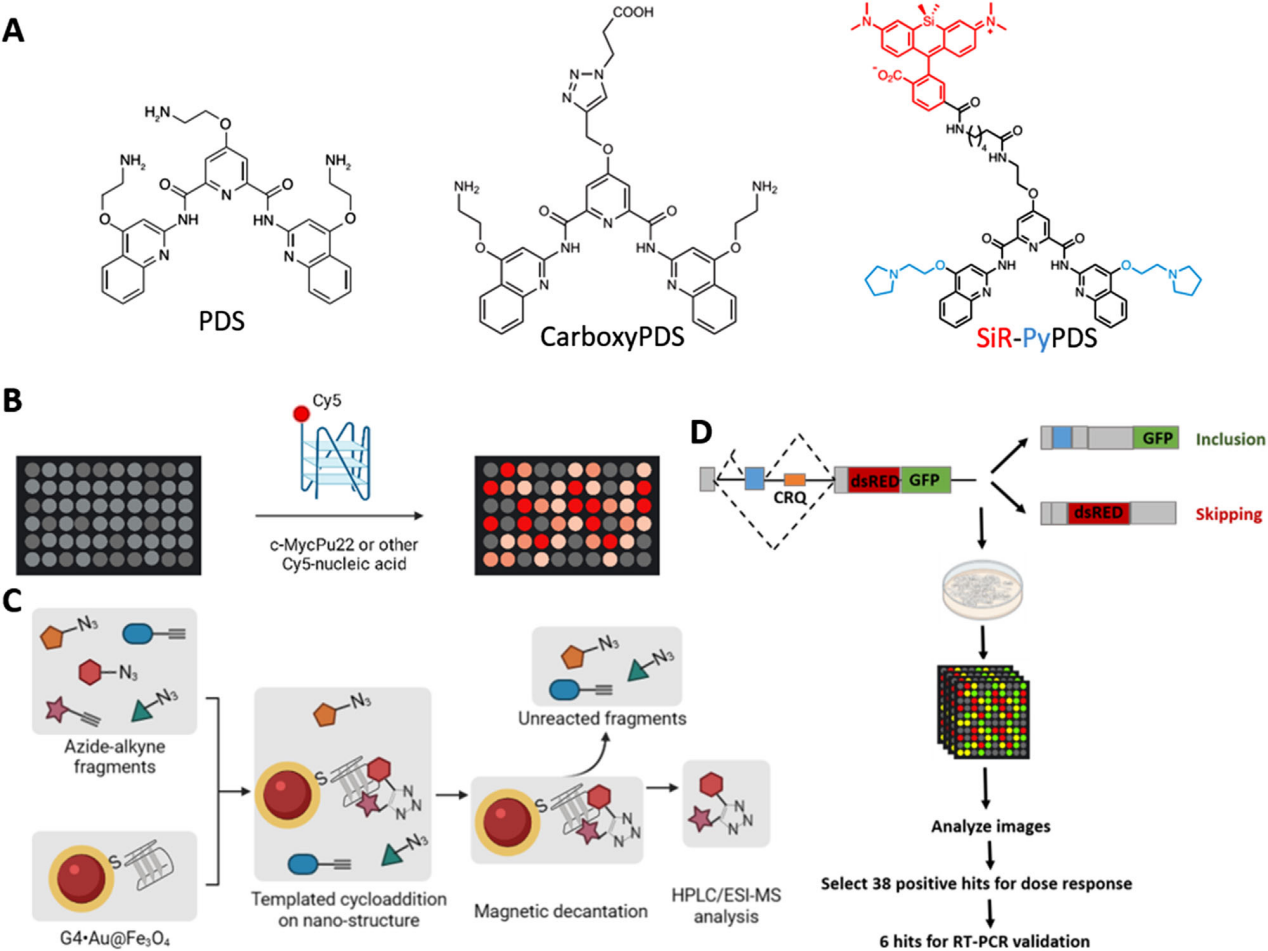
G‐quadruplex probes and the screening technologies. (A) Several representative G4 ligands. (B) Schematics of small molecular microarray (SMM) for screening G4 ligands.^[^ ^69^ ^]^ (C) Schematics of nano‐target guided synthesis (Nano‐TGS) for screening G4 ligands.^[^ ^70^ ^]^ (D) Schematics of confocal high‐content imaging system for screening small molecules that can affect RNA alternative splicing by binding to the G4 structures.^[^ ^71^ ^]^

The detection and biochemical targeting of intracellular G4s depend on the chemical tools for probing G4s with high specificity. Small molecule ligands and antibodies are the major types of widely used G4 probes. At present, a series of G4 ligands have been developed. These ligands have the following three characteristics in common (Figure 2A): (1) have an aromatic planar structure to form a π–π stacking interaction with the G4 structures; (2) have a positive charge or a basic group to interact with G4 loops or grooves; (3) have a certain three‐dimensional structure to avoid binding to double‐stranded DNA and losing specificity. According to the molecular structure, there have been seven categories of G4 ligands: anthraquinoes, benzo[a]phenoxazines, indoloquinolines, naphthalene diimides, phenatrolines, quinazolines, and telomestatin derivatives, which has been elaborated elsewhere.^[^ ^60^ ^]^ Currently applicable G4 ligands have been collected in the G4LDB (G4 ligand database, https://www.g4ldb.com).^[^ ^61^ ^]^

The most widely used G4 ligands are pyridostatin (PDS) and its derivatives (Figure 2A). PDS has high binding affinity to G4s and can bind to all topological types of G4s with little bias.^[^ ^62^ ^]^ Thus, PDS has been widely applicated in biophysical characterization and biochemical targeting of G4s. CarboxyPDS has higher binding affinity to RNA G4s than DNA G4s, which can be very useful in the detection of RNA G4s.^[^ ^63^, ^64^ ^]^ Another special G4 fluorescent probe derived from PDS is SiR‐PyPDS, which cannot induce unfolded G4‐forming sequences to fold into G4 structures at low concentration (20 nM).^[^ ^65^ ^]^ This feature is very necessary in the detection of intracellular G4s with its intact folding state.

In addition to the small molecule ligands, G4 antibodies are also a powerful tool for G4 probing, including BG4,^[^ ^66^ ^]^ 1H6,^[^ ^67^ ^]^ and D1.^[^ ^68^ ^]^ These antibodies have played an important role in G4 imaging and sequencing due to its high binding affinity and selectivity to the G4 structures.

### 3.2 Methods for screening G4 probes

Given the importance of G4 probes, it is worth to introduce the methods for the design and optimization of G4 probes, which are critical in the future development of G4 bioanalysis and biochemical targeting toolkits. The G4 antibodies are screened with the common phase display method.^[^ ^72^ ^]^ In contrast, the methods for screening small molecule ligands are more complicated.

Many high‐throughput screening methods for G4 ligands have been developed to obtain and improve the G4 probes. For instance, the small molecular microarray (SMM) has been used to screen ligands of the MYC promoter G4^[^ ^69^ ^]^ (Figure 2B). Firstly, a pool of 20,000 small molecules was covalently attached to different areas of the glass plate through isocyanate surface chemistry. Next, the Cy5‐labeled MYC G4 oligonucleotides were incubated with the glass plate, and then washed with buffer. Finally, the ligand with high affinity can be identified by comparing the fluorescence intensity on the glass plate. Through SMM, a specific ligand for MYC G4 was obtained. However, this method is complicated and costly.

Alternatively, Flusberg *et al*. reported an automated ligand identification system (ALIS) for screening the ligands of MYC G4.^[^ ^73^ ^]^ ALIS utilized mass spectrometry to identify the G4 molecules, the ligands, as well as the G4‐ligand complexes. The binding affinity of ligands can be calculated by the ratio between the signal intensity of G4‐ligand complexes and that of the ligands. ALIS has the advantages of being fast and easy to operate. ALIS does not need to immobilize small molecules on the substrate and label G4 molecules, and only needs a small amount of reagents. In this way, as many as 500,000 molecules can be screened with an ALIS system in only one day.

Another powerful method is the nano‐target guided synthesis (Nano‐TGS)^[^ ^70^ ^]^ (Figure 2C), in which the screening is completed during the synthesis process. Alkyne building blocks and azide building blocks were incubated with magnetic beads, with which MYC G4 molecules were conjugated. Through G4 guided combination chemistry, G4 ligands were generated and purified via the magnetic beads, which were further identified by HPLC/ESI‐MS. The G4‐conjugated magnetic beads can be reused for up to five reaction cycles, which is cost‐effective. The screened molecule has been proved to inhibit MYC gene transcription in vivo. However, only a small pool of less than ten building blocks can be used in the screening process, resulting in a relatively low throughput.

G4 probes serve as not only sensors for detecting G4 structures, but also biochemical regulating tools for modulation of G4 biofunctions. In this way, it is also necessary to screen the G4 ligands according to their biological effect in living cells, in addition to obtain high‐affinity G4 ligands. For example, Zhang *et al*. used the confocal high‐content imaging system to screen out small molecules that can affect RNA alternative splicing by binding to the G4 structures^[^ ^71^ ^]^ (Figure 2D). A dual‐color splicing reporter contains a G4 structure proximal to the splicing site. When the RNA is spliced in different ways, different ratios of two fluorescent proteins will generate. Using 384‐well plates and the automatic confocal microscopy, the regulatory effect of the ligands in each well can be obtained. This method is beneficial to directly obtain small molecule drugs that can be used to regulate biological process in living cells via G4s. In the future application of this method, it is important to design the fluorescent reporter system for other G4‐mediated bioprocess other than splicing, such as transcription, translation, and so on. To our best knowledge, this is the only one method for screening G4 ligands according to their bioregulatory results. Given that G4 ligands can act in many different biological processes, it is important to obtain G4 ligands that only act in a certain specific pathway. Therefore, more new technologies in this field are under demand.

## 4 VISUALIZING INTRACELLULAR DNA AND RNA G4s

### 4.1 Cell imaging of intracellular G4s

**FIGURE 3:**
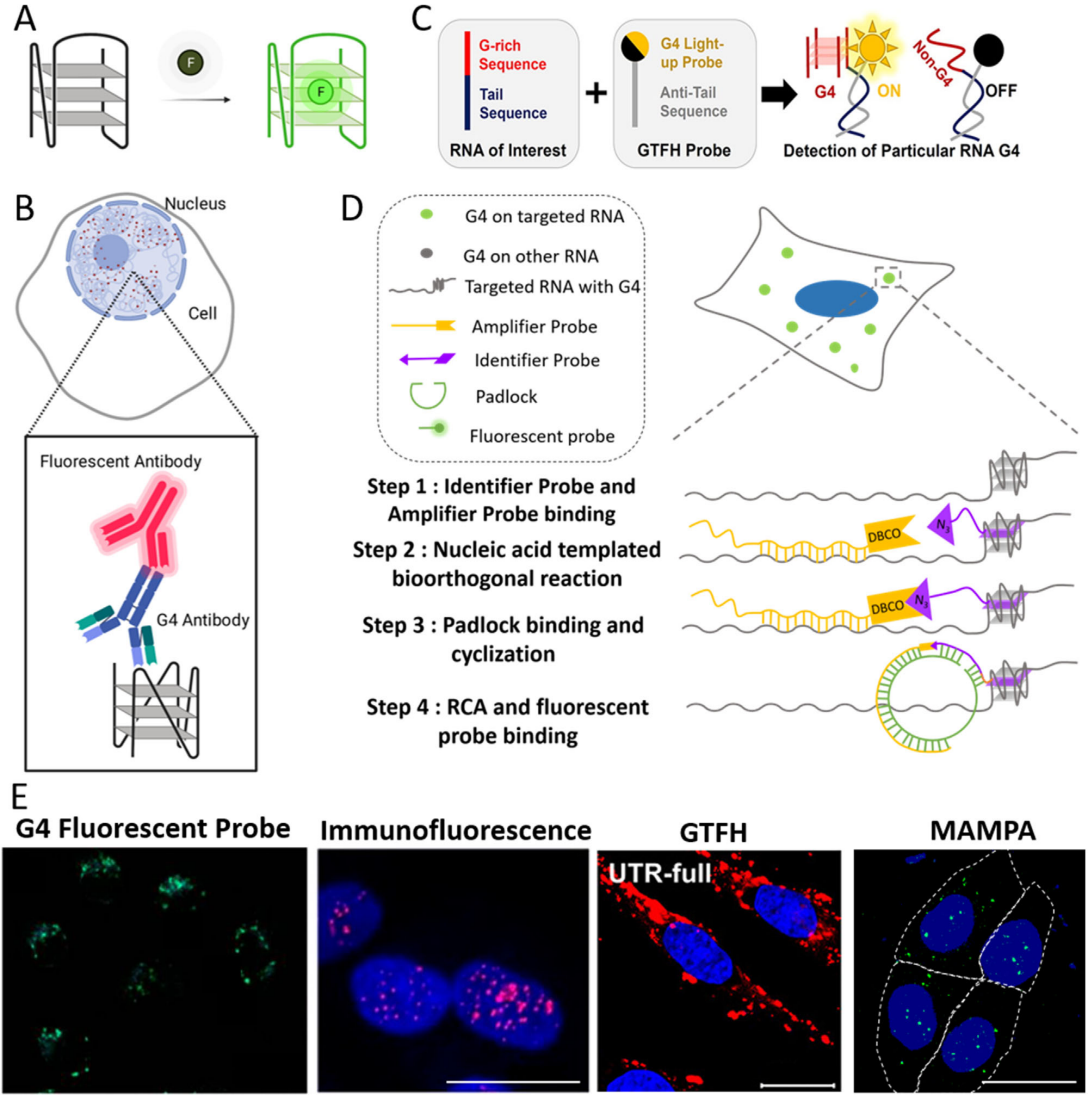
Visualizing intracellular DNA and RNA G4s. (A) The principle of G4 fluorescent probes. (B) Visualization of nuclear G4s by immunofluorescence using G4 antibodies.^[^ ^66^ ^]^ (C) The principle of G4‐triggered fluorogenic hybridization (GTFH) probe for imaging G4 in a specific gene. Reproduced with permission.^[^ ^84^ ^]^ Copyright 2016, American Chemical Society. (D) Module assembled multifunctional probes assay (MAMPA) for visualizing monogenetic G4s in fixed cells. Reproduced with permission.^[^ ^86^ ^]^ Copyright 2021, Wiley‐VCH GmbH. (E) Comparison of different G4 imaging methods. G4 Fluorescent probe panel: Reproduced with permission.^[^ ^80^ ^]^ Copyright 2015, American Chemical Society. Immunofluorescence panel: Reproduced with permission.^[^ ^98^ ^]^ Copyright 2020, American Chemical Society. GTFH panel: Reproduced with permission.^[^ ^84^ ^]^ Copyright 2016, American Chemical Society

In recent years, fluorescent imaging methods for detecting G4s in the cells have achieved great progress (Table 1). Many small‐molecule fluorescent probes of G4s have been developed to visualize G4s in living cells^[^ ^74^, ^75^ ^]^ (Figure 3A). These probes are commonly derived from G4 ligands.^[^ ^75^ ^]^ The key property of G4 probes lies in its selectivity to the G4 structures with turn‐on fluorescence emission. In other words, upon binding to DNA and/or RNA G4s, the probes display significantly increased fluorescence relative to background signal. No obvious fluorescence changes should be observed when the probes are incubated with other types of nucleic acids, such as hairpins, single strands, and duplexes. Representative fluorescent probes of DNA G4s include ATPD,^[^ ^76^ ^]^ TSQ1,^[^ ^77^ ^]^ DAOTA‐M2,^[^ ^78^ ^]^ and IMT.^[^ ^79^ ^]^ Turning to RNA G4s, representative fluorescent probes are NaphthoTASQ,^[^ ^80^ ^]^ CyT,^[^ ^81^ ^]^ QUMA‐1,^[^ ^82^ ^]^ and PhenDC3‐Cy5.^[^ ^83^ ^]^ These probes have all been displayed to image G4s in living cells.

**TABLE 1:** Representative G4 imaging methods

**Methodology**	**Technology**	**Uses and advantages**	**Limitation**	**Single‐molecule imaging**	**Recognizing monogenetic G4s**	**Ref**.
Small‐molecule fluorescent probes	ATPD, TSQ1, DAOTA‐M2, IMT	Visualizing DNA G4s in living cells.	Cannot image G4s at single‐molecular level. Cannot recognize monogenetic G4s.	No	No	[76, 77, 78, 79]
	NaphthoTASQ, CyT, QUMA‐1, PhenDC3‐Cy5	Visualizing RNA G4s in living cells.		No	No	[80, 81, 82, 83]
	SiR‐PyPDS	Detecting G4s without influencing their original folding state. Imaging G4s at single‐molecular level in living cells.	Cannot recognize monogenetic G4s.	Yes	No	[65]
Immunofluorescence	Immunofluorescence imaging of DNA G4s	Visualizing the overall DNA or RNA G4s folding state in fixed cells.	Cannot image G4s at single‐molecular level. Cannot recognize monogenetic G4s.	No	No	[63]
	Immunofluorescence imaging of RNA G4s					[66]
Imaging G4s on individual genes	GTFH	Imaging the G4 motif of NRAS mRNA in living cells.	Without a signal amplification strategy, cannot image G4s at single‐molecular level.	No	Yes	[84]
	MAMPA	Imaging monogenetic G4s in fixed cells.	The throughput is limited. Only one RNA G4 site can be visualized at one time. Cannot be applied in living cells.	Yes	Yes	[86]

Fluorescent probes of G4s for use in living cells need to have high specificity, low cytotoxicity, and strong cell permeability. It is particularly important that the G4 fluorescent probes should not induce the formation of additional G4 structures during the detection process, so as to ensure that the observed signal is only generated by the G4s already folded in the cells. However, the ideal probe that can meet all requirements has not yet been reported. The major unsolved problem is that many G4 probes can induce the formation of G4s. Recently, Di Antonio *et al*. reported an advanced DNA G4 fluorescent probe, SiR‐PyPDS, that detected G4s in vivo without disordering their folding dynamics.^[^ ^65^ ^]^ SiR‐PyPDS was obtained by conjugating PyPDS with the far‐red fluorophore silicon‐rhodamine. PyPDS is an analog of the widely used G4 ligand PDS, and its binding affinity is slightly weaker than PDS. At its work concentration of 20 nM, SiR‐PyPDS was proved to not induce more G4 formation. Combined with single‐molecule and real‐time microscopy, SiR‐PyPDS enabled exact detection of the repertoire of intact G4s in living cells.

An alternative G4 imaging method is immunofluorescence based on G4 antibodies. BG4 is the most widely used G4 antibody (Figure 3B), which can bind to DNA and RNA G4 with high specificity and high affinity (*K*_d_ = 1–15 nM).^[^ ^66^ ^]^ Immunofluorescence imaging of DNA and RNA G4s has been realized in fixed mammal cells.^[^ ^63^, ^66^ ^]^ This method enables the measurement of the overall G4 folding state in dynamic cellular processes, such as the transformation of chromosomes during mitosis. However, whether this method can detect a single G4 has not been effectively confirmed. The number of fluorescent scatters in the nucleus is relatively small, far lower than the theoretical number of G4s contained in various genes. In other words, immunofluorescence may only visualize G4 structures that are clustered together in space. Moreover, G4 antibodies may not be able to bind to all possible G4 configurations,^[^ ^25^ ^]^ which restricts the detection efficiency.

### 4.2 Imaging G4s on individual genes

The above‐mentioned imaging methods have promoted the understanding of the biological roles of G4s, but still have some shortcomings. The major problem of the imaging methods is that the entirety of intracellular G4s always been probed at the same time. Regardless of small molecule ligands or antibodies, they can only distinguish G4s from other nucleic acid types, such as double strands, single strands, hairpins, and so on, but cannot distinguish the G4s with different sequence contexts in various genes. In actual, all G4s have the consensus feature of stacked G‐quartets, while the differences only lie on the loop regions. The impact of these differences on the overall global similarity of most G4s is very weak, resulting in difficulty in detecting the single G4 motif. It was unsuccessful to screen specific probes from a small pool of three different G4s,^[^ ^85^ ^]^ let alone differentiate the G4 of an individual gene among the entire genome. Therefore, how to achieve the specific detection of the G4 on a single gene is the ultimate goal for this field.

There has already been some progress about the imaging of monogenic G4s. Chen *et al*. reported the G4‐triggered fluorogenic hybridization (GTFH) probe, ISCH‐nras1.^[^ ^84^ ^]^ This probe consists of two parts, one is a hybridization probe targeted to the proximity sequence near the G4 motif of NRAS mRNA, and the other is a G4 light‐up probe, of which the fluorescence can be enhanced by adjacent G4s (Figure 3C). This probe has been examined to specifically label NRAS G4s in living cells, and become the first applicable probe for RNA G4s of an individual gene. However, ISCH‐nras1 can only enable the visualization of G4s of exogenously transfected NRAS mRNA, resulting from its limited fluorescent intensity in the cell context and the lack of a signal amplification strategy. The application of this probe for studying monogenic G4s is largely restricted by this shortcoming.

Recently, our group developed module assembled multifunctional probes assay(MAMPA) for visualizing monogenetic G4s in fixed cells^[^ ^86^ ^]^ (Figure 3D). In this method, two modular probes separately identify G4 structures and adjacent RNA sequences. The ID‐probe consists of a G4 ligand CarboxyPDS and an azide conjugated polyadenylic acid backbone. The Amp‐probe is a 5′‐dibenzocyclooctyne (5′‐DBCO) modified hybridization probe to recognize the targeted RNA by complementation. Nucleic acid templated bioorthogonal click reaction chemically ligates the two probes, upon the targeted monogenic G4s are recognized. Rolling circle amplification (RCA) is utilized to recognize the assembled probes and amplify the fluorescent signal. It has been demonstrated that the detection of RNA G4s in high or low expression genes could be achieved by MAMPA. Till now, this has become the only one method that is capable of imaging the G4s of a single endogenous gene. Compared to other G4 imaging methods, MAMPA exhibits the most accuracy (Figure 3E). However, after the cell is fixed, the dynamic and transient folding of G4s cannot be captured, which limited its application. It can be expected that monogenic G4 bioanalytical methods with better performance will be increasingly developed in the future.

## 5 HIGH‐THROUGHPUT SEQUENCING METHODS FOR DETECTING G4s

The cell imaging methods of G4s open a way to observe the in situ folding state of G4 structures in the cellular context, which is beneficial to directly monitor the changes of the whole pool of intracellular G4s in the dynamic biological processes. However, these methods cannot provide the detailed sequences of intracellular G4s, which are critical to derivate the distribution and localization of observed G4s on the genome and transcriptome. Using bioinformatical methods, the consensus G4 formula has been mapped to the genome and transcriptome of human, mouse, and other species, and more than 1 million potential G4‐forming sequences were obtained.^[^ ^32^, ^33^ ^]^ Importantly, not all potential G4‐forming sequences can be folded into a quadruplex structure in the cells. In addition, the formation of nucleic acid secondary structures can be regulated by the DNA‐binding proteins and RNA‐binding proteins in every specific cellular context, which further increases the complexity of the intracellular folding state of the G4 structures. Therefore, it is necessary to detect the folding state and G4‐protein interaction of the intracellular G4 entirety with high‐throughput sequencing methods. Representative G4 high‐throughput sequencing methods are listed in Table 2.

**TABLE 2:** Representative G4 high‐throughput sequencing methods

**Methodology**	**Technology**	**Uses and advantages**	**Limitation**	**Ref**.
Detecting DNA G4s in cellular genome	G4‐seq	Identifying the observable G4s in the human genome.	Cannot distinguish G4s in proximity.	[87, 91]
	G4‐Miner	Mapping G4s in the genome with simple operation and improved accuracy.	Positive detection rate is low.	[95]
Detecting RNA G4s in cellular transcriptome	rG4‐seq	Identifying the formation and stability of RNA G4s.	Cannot reflect the real folding state of RNA G4s in vivo.	[92]
	RT Stop Profiling	Mapping RNA G4s in vivo.	Positive detection rate is low due to the unexpected reaction on the G bases of folded G4s.	[96]
	G4RP‐seq	Mapping transcriptomic G4s in the physiological state.	The false positive signal can be enriched due to indirect RNA interactions.	[93]
Detecting G4s in chromatins	G4 ChIP‐seq	Mapping G4s in chromatins.	Low signal‐to‐noise ratio.	[34, 99]
	G4 CUT&Tag	Mapping Chromatin G4s with high resolution and low background with small number of cells.	The G4 antibodies may have bias in binding G4s in different regions of the genome, resulting in bad reproducibility.	[94]

### 5.1 Detecting DNA G4s in cellular genome

**FIGURE 4:**
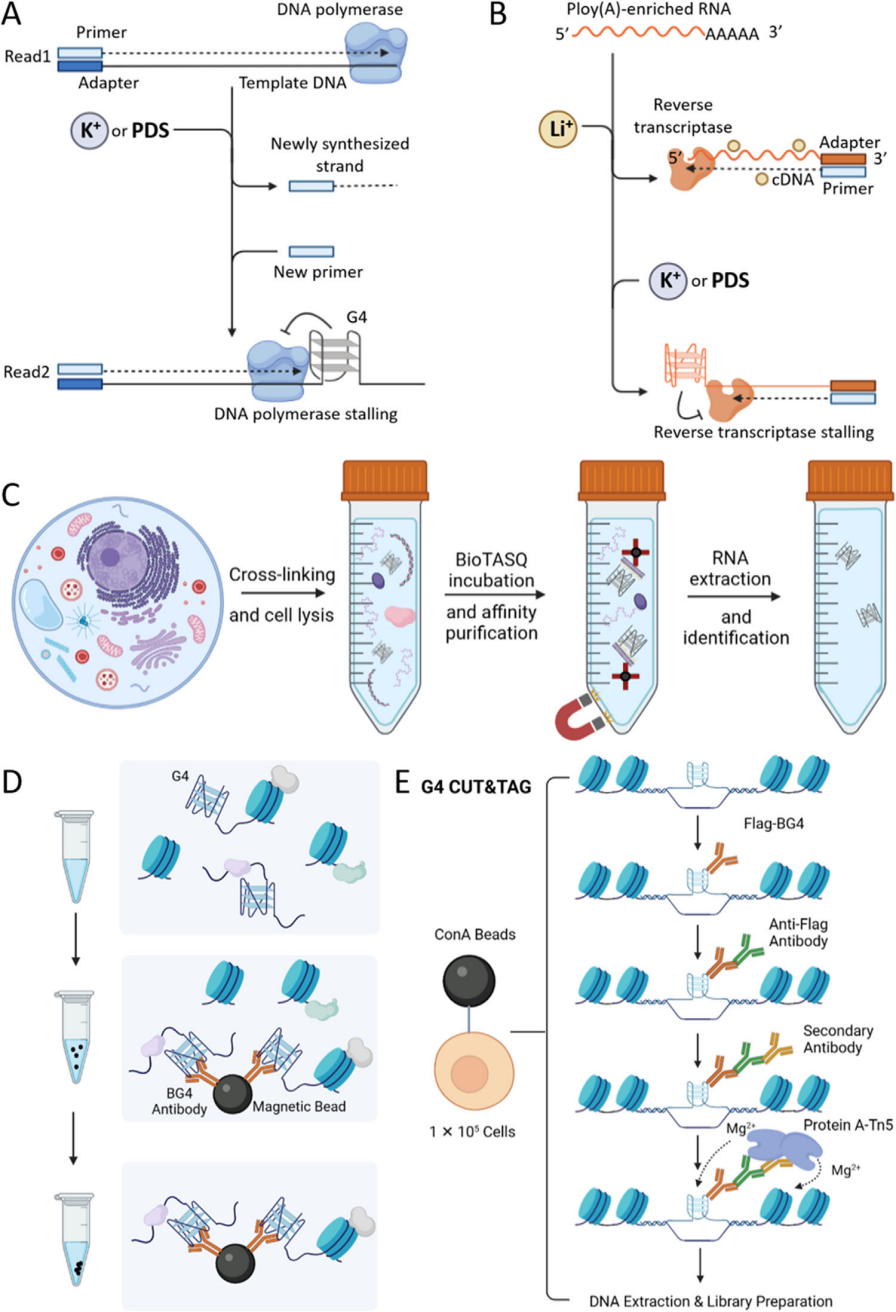
G‐quadruplex high‐throughput sequencing methods. (A) G4‐seq.^[^ ^87^ ^]^ (B) rG4‐seq.^[^ ^92^ ^]^ (C) G4RP‐seq.^[^ ^93^ ^]^ (D) G4 ChIP‐seq.^[^ ^34^ ^]^ (E) G4 Cut&Tag.^[^ ^94^ ^]^

Investigating the sequences that can fold into G4 structures has been achieved by a polymerase stalling method.^[^ ^87^ ^]^ As is widely known, G4s can be stabilized by monovalent ions.^[^ ^88^ ^]^ Based on the match between the ion radius and the G4 topological structures, the relative stabilizing ability of the common monovalent ions is K^+^ > Na^+^ > > Li^+^. Therefore, with the presence of a high concentration of K^+^, the highly stabilized G4s on the template strand of DNA can stall the elongation of the DNA polymerase. This principle has been examined for detecting G4s,^[^ ^89^ ^]^ but only one G4‐forming oligonucleotide can be detected at a time. Inspired by this method, the polymerase stalling principle has also been applied in the Illumina sequencing method, which is called G4‐seq^[^ ^87^ ^]^ (Figure 4A). During the “sequencing by synthesis” process, the stalling caused by G4s results in a sharp drop of the Phred quality scores^[^ ^90^ ^]^ after the G4 site. In fact, during the sequencing process, there are always some reads with lower sequencing quality, not all of them result from G4 structures. To eliminate the interference of random drop of sequencing quality, read 1 and read 2 processes of pair‐end sequencing were separately treated with Na^+^ and K^+^ (or G4 ligand PDS). A sequencing buffer with Na^+^ ions was used in the read 1 process, in which the G4s cannot fold and the accuracy of DNA sequence recognition can be ensured. Then, a sequencing buffer containing K^+^ was used in the read 2 process, generating drastically reduced sequencing quality scores on the same clusters. In this way, the sequence capable of folding into G4s can be accurately identified.

G4‐seq has been applied to identify the observable G4s in the human genome. Only 60–70% of the predicted G4s in the human genome can be observed experimentally to be folded. There are in total 716,310 different observable G4s discovered by G4‐seq, of which more than 70% are non‐canonical G4 structures that cannot be predicted by computational algorithm. The non‐canonical G4s include long loops with more than 7 bases, bulges with discontinuous G‐tracts regions, and two‐tetrad G4s. These results indicate that the changes in the local sequence context in the genome have very complex affection on the formation and stability of the G4 structures. Therefore, computational algorithms always over‐predict or under‐predict many G4 structures, making it difficult to accurately identify the formation of G4s in the genome. The G4‐seq method has been further optimized and applied to the genomic G4 sequencing of 12 species,^[^ ^91^ ^]^ which has provided important insights for the formation and distribution of genomic G4s.

However, the G4‐seq method identifies G4s based on the polymerase stalling site, resulting in insufficient spatial resolution. The G4s in proximity may not be effectively distinguished. Moreover, G4‐seq relies on customized Illumina sequencing reagents with the flexibility of adding different monovalent ions (not commercialized), which limited the usage of this method by the other groups. Recently, the G4‐Miner method was reported to be used to detect genomic G4s through a novel principle.^[^ ^95^ ^]^ In the standard whole‐genome sequencing process of Illumina sequencing, the reaction buffer without K^+^ cannot lead to the formation of stable G4s, but the existing unstable G4s can still cause abnormal fluctuations of the Phred quality scores. Through capturing the subtle changes in the sequencing quality of these sites with computational algorithm, the G4 structures can be directly predicted. G4‐Miner detected 736,689 G4 sites in the human genome, 89% of which were consistent with the results obtained by G4‐seq. Although G4‐Miner is simple and accurate, its positive detection rate is still somewhat low, compared to the improved G4‐seq method. Therefore, it is necessary to develop more genomic G4 sequencing methods to promote the advancement of DNA G4 biology.

### 5.2 Detecting RNA G4s in cellular transcriptome

Turning to the detection of RNA G4s in the transcriptome, the progress of this field has kept increasing. rG4‐seq is the first method for sequencing transcriptomic G4s,^[^ ^92^ ^]^ with the similar principle of G4‐seq (Figure 4B). The fragmented mRNA from the cell transcriptome was used in the reverse transcription. Under the conditions of high concentration of K^+^ and/or PDS, the reverse transcriptase can be blocked by G4s, resulting in frequently stalled sites at the upstream of G4s. This specific feature can be utilized to identify RNA G4s from the sequencing data.

rG4‐seq has shown insightful results about intracellular RNA G4s. In the transcriptome of HeLa cells, 3845 RNA G4s were identified under K^+^ conditions and 13,423 under PDS conditions. Transcriptomic G4s also have the long loop, the bulge, and two G‐tetra motifs, which is similar to genomic G4s. Unlike the genomic G4s, the transcriptomic G4s of the same species have significant differences in different cell states and cell types. Therefore, RNA G4 sequencing for different cell lines, and especially disease samples, is of great significance for revealing the mechanism of RNA G4 in complex biological processes.

Although the rG4‐seq method can identify the formation and stability of transcriptomic G4s with high throughput, it may have bias in detecting the folding state of G4s. In fact, the G4s that remain unfolded in vivo, may be induced to fold into G4s during the experimental process. In vivo RNA G4 sequencing has been attempted by Guo *et al*. through RT Stop Profiling method,^[^ ^96^ ^]^ which is also depended on the polymerase stalling principle. Dimethyl sulfate (DMS) and 2‐methylnicotinic acid imidazolide (NAI) are widely used to identify the secondary structures of RNA. After treatment by these reagents in the living cells, the unpaired and unconstrained nucleotides are kinetically more susceptible to be reacted and cannot form any secondary structures later. In this way, if the G4‐forming sequences are unfolded in the living cells, the associated G bases are methylated or acylated, and subsequently the RNA does not form G4 structures and cause polymerase stalling. However, this method may also cause some bias due to the unexpected reaction on the G bases of folded G4s, which has been discussed in detail elsewhere.^[^ ^97^ ^]^ In addition, G4RP‐seq is a more accurate method to detect transcriptomic G4s in the physiological state^[^ ^93^ ^]^ (Figure 4C). Firstly, the cells are cross‐linked with formaldehyde to halt biological processes and fix transient structural interactions, then the G4 containing RNA is captured by biotin‐modified G4 ligand TASQ (BioTASQ), and finally enriched with streptavidin magnetic beads. Through this method, the G4s in both mRNA and long non‐coding RNA can be detected, which expands the understanding of intracellular RNA G4s. Because the cross‐linking process fixes the secondary structures, it will not induce the unfolded sequences to form G4s during the detection process. However, the false positive RNA can be enriched through direct or indirect RNA interactions after cross‐linked, making it difficult to identify G4s from the less abundant RNA. Therefore, it is urgent to develop more methods that can accurately detect transcriptomic G4s in living cells.

### 5.3 Detecting G4s in chromatins

In living cells, DNA binds to histones to form nucleosomes, which densely fold into chromatins. Chromatin DNA does not completely exist in the form of double‐stranded DNA. Some secondary nucleic acid structures, including G4 and i‐motif, have been confirmed to exist in chromatin DNA.^[^ ^98^ ^]^ Interestingly, among all the observed G4s in mammal genome, only a small percentage can fold into G4 structures in chromatin DNA. Therefore, the distribution and localization of these chromatin G4s may play an important role in gene expression regulation. Profiling of chromatin G4s has been realized by ChIP‐seq with G4 antibody BG4^[^ ^34^, ^99^ ^]^ (Figure 4D). The landscape of chromatin G4s further confirmed the relationship between G4s and transcription regulation. In K562 cell line, there are more than 8000 high‐confidence G4 sites, which are highly enriched in transcriptionally active region. In addition, ChIP‐seq of DNA G4 binding proteins (G4BPs) can indirectly identify chromatin G4s. G4 binding transcription factors, including Rif1,^[^ ^12^ ^]^ XPD, XPB,^[^ ^31^ ^]^ and so on, have been found to be abundantly enriched in chromatin G4 locus, proving that G4s exist in chromatin DNA and involve in transcription regulation.

Cleavage Under Targets and Tagmentation (CUT&Tag) is a recently invented method for efficiently profiling chromatin DNA binding maps.^[^ ^100^ ^]^ Compared with ChIP‐seq, it has high resolution and low background, and requires only a small number of cells to achieve high‐sensitivity detection. Cut&Tag has been used in chromatin G4 detection^[^ ^94^ ^]^ (Figure 4E). However, the performance of Cut&Tag for G4s is unsatisfactory. G4 Cut&Tag of mESC cells from different groups showed significantly different results, ranging from one to ten thousand of peaks.^[^ ^94^ ^]^ This may be because the G4 locus is also a high GC ratio locus, which can inhibit PCR amplification during the preparation of sequencing library.^[^ ^101^ ^]^

These techniques for mapping the chromatin G4s, have promoted the exploration of the functions of intracellular G4s. The chromatin G4s have been demonstrated to be a transcription factor hub, which plays a key role in the recruitment of transcription factors and the assembly of RNA Pol II.^[^ ^10^, ^102^ ^]^ G4s have been proved to shape the cell specific transcriptome.^[^ ^35^ ^]^ Furthermore, G4s have also been confirmed to mold DNA methylomes and inhibit the activity of DNA methyltransferase DNMT1.^[^ ^38^ ^]^ However, ChIP‐seq and Cut&Tag all depend on G4 antibodies, which are expected to have high binding affinity and high specificity. Among the known G4 antibodies, BG4 has the best performance, but is likely to have some bias in the detection of chromatin G4s.^[^ ^25^ ^]^ Recently, a new G4‐binding peptide G4P has been reported to detect more G4 peaks at high resolution with ChIP‐seq.^[^ ^103^ ^]^ A greater proportion of peaks are perfectly matched with the observed genomic G4s. G4P is expected to become a better chemical tool for improving our understanding of chromatin G4s.

## 6 PROFILING THE INTERACTION BETWEEN G4s AND PROTEINS

G4BPs are a series of proteins that can bind to G4 structures. The known G4BPs have been reported in G4 DNA/RNA Interacting Protein Database (G4IPDB, http://bsbe.iiti.ac.in/bsbe/ipdb/index.php). It has been demonstrated that many G4‐associated biological processes are mediated by G4BPs. G4BPs participate in G4‐associated processes with the following three mechanisms: (1) G4BPs work directly after binding with G4s without unfolding G4 structures; (2) G4BPs bind to and unfold G4 structures to reduce the hindrance of G4s in biological processes such as transcription and translation;^[^ ^49^ ^]^ (3) G4BPs recruit other proteins after binding with G4s, regarding G4 structures as scaffolds.^[^ ^104^ ^]^ In order to generate a robust molecular understanding of intracellular G4s, it is necessary to uncover which proteins can bind to G4 structures with high affinity and specificity, what is the interaction network between G4BPs and G4s in living cells, and how these G4BPs participate in biological processes. Therefore, a series of methods have been developed to analyze G4BPs (Table 3).

**TABLE 3:** Representative G4 proteomic methods

**Methodology**	**Technology**	**Uses and advantages**	**Limitation**	**Ref**.
Pull‐down methods	Stable isotope labeling by amino acids in cell culture (SILAC)	Identifies G4BPs binding with the G4 site of the human telomere and the promoters of cMYC and cKIT gene. Provides the relationship between G4BPs and a specific DNA G4 site.	Cannot reveal the in situ G4‐protein binding state in living cells. The throughput for G4 sequences is limited. Only one G4 sequence a time	[106]
	A pull‐down method combined with isotope‐labeled mass spectrometry	Characterizes the binding affinity between G4s and G4BPs.	Compared with the traditional method of measuring the binding affinity, ELISA, the complexity of operation and the cost is higher.	[109]
	RNA G4 pull‐down method	Identifies G4BPs binding with the G4 site of NRAS 5′UTR region. Provides the relationship between G4BPs and a specific RNA G4 site.	Cannot reveal the in situ G4‐protein binding state in living cells. The throughput for G4 sequences is limited. Only one G4 sequence a time.	[110]
In situ labeling methods	Co‐binding‐mediated protein profiling (CMPP)	Reveals the type and abundance of G4BPs binding with G4s in living cells.	Cannot provide the relationship between G4BP and the specific G4 containing genes.	[107]
	G4 ligand‐mediated cross‐linking and pull‐down (G4‐LIM‐CAP)	Reveals the type and abundance of G4BPs binding with G4s in living cells.	Cannot provide the relationship between G4BP and the specific G4 containing genes.	[111]

In the early stage of research on G4BPs, in vitro methods, such as electrophoretic mobility shift assay, surface plasmon resonance, and DMS footprinting, were used to study the interaction between one single G4BP and one single G4‐forming sequence.^[^ ^105^ ^]^ These methods can help to understand the binding affinity between G4BPs and G4s in vitro. However, these methods have low throughput, and cannot reflect the in situ G4‐protein binding state in living cells. Therefore, it is necessary to develop high‐throughput methods to characterize G4BPs.

### 6.1 Pulldown methods for G4BPs

**FIGURE 5:**
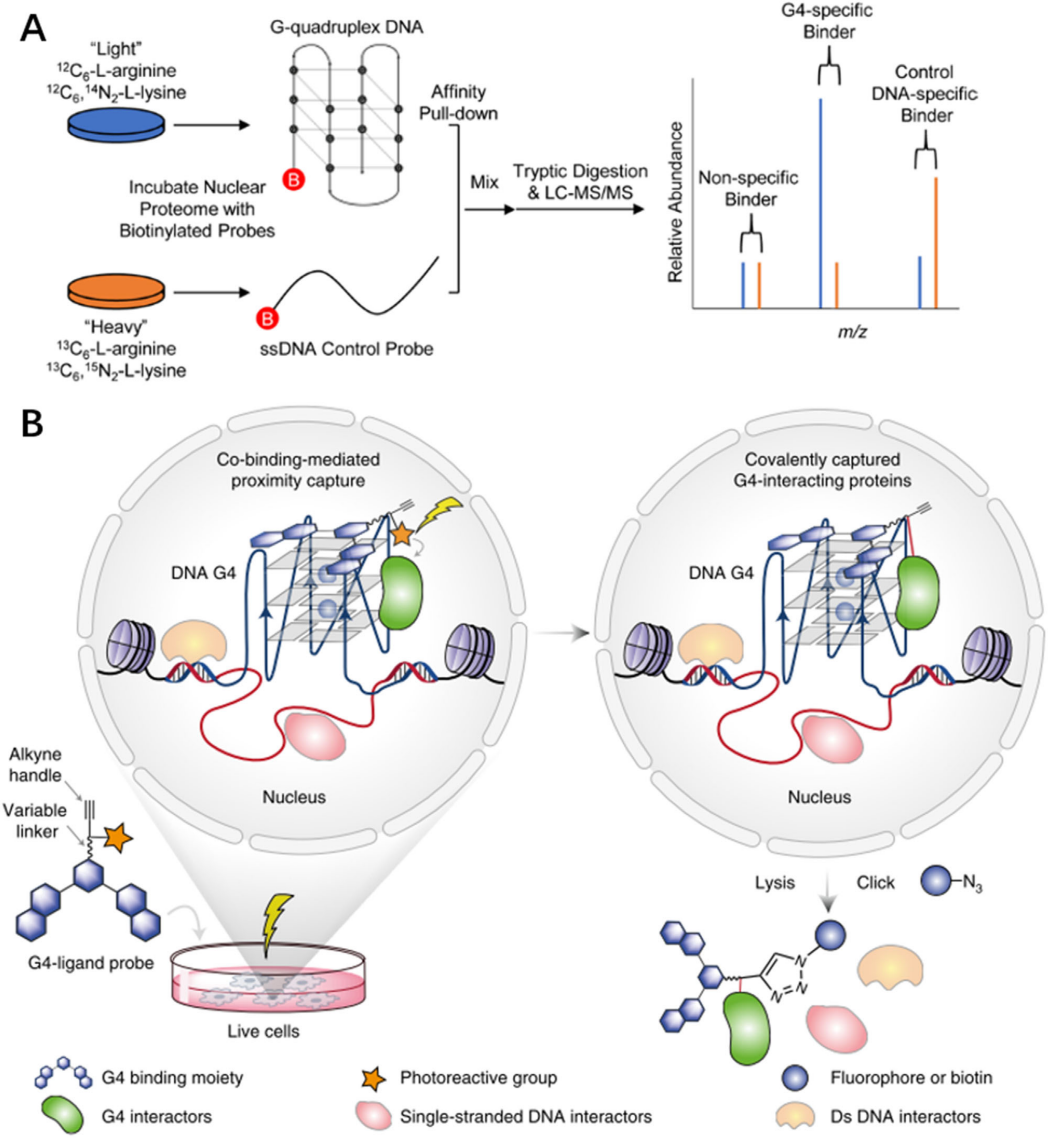
Methods for profiling the G4‐protein interactions. (A) Quantitative mass spectrometry based on SILAC (stable isotope labeling by amino acids in cell culture) to identify G4BPs. Reproduced with permission.^[^ ^106^ ^]^ Copyright 2017, American Chemical Society. (B) Co‐binding‐mediated protein profiling (CMPP) for detecting G4BPs in living cells. Reproduced with permission.^[^ ^107^ ^]^ Copyright 2015, The Authors

Mass spectrometer is widely used to identify proteins, which is called high‐throughput proteomics. Proteomic technologies have been applicated to discover and characterize G4BPs. Williams *et al*. used quantitative mass spectrometry based on SILAC (stable isotope labeling by amino acids in cell culture) to identify G4BPs^[^ ^106^ ^]^ (Figure 5A), which was the first work on G4 binding proteome. In this method, the G4BPs were pulled down from nucleoprotein extracts by biotin‐labelled G4 oligonucleotides, and characterized by LC‐MS/MS. Using this method, many G4BPs, including SLIRP, BmLARK,^[^ ^108^ ^]^ and so on, were discovered. Beyond profiling the landscape of G4BPs, pull‐down methods can also be used to measure the binding affinity of G4BPs. For instance, Makowski *et al*. developed a pull‐down method combined with isotope‐labeled mass spectrometry to characterize the binding affinity between G4s and G4BPs.^[^ ^109^ ^]^ When the cell lysate is incubated with G4 oligonucleotides at different concentrations, the amount of G4‐protein complexes will vary according to the binding affinity. The samples were labeled with TMT (tandem mass tag) to realize multiplexing relative quantification and obtain the binding curves. Similar to the DNA G4 pull‐down method, Herdy *et al*. developed the RNA G4 pull‐down method to study the G4BPs located at the G4 sites of the proto‐oncogene NRAS 5′UTR region.^[^ ^110^ ^]^ Through this method, 80 proteins were identified to specifically bind to NRAS RNA G4, and five new G4BPs were discovered, including DDX3X, DDX5, GRSF1, and NSU5.

### 6.2 **In situ** labeling G4BPs in living cells

The above‐mentioned pull‐down methods are easy to operate, but they are performed in the cell lysate, which cannot reflect the in situ G4‐protein binding state. In other words, proteins that have the potential to bind to G4s in vitro may not always bind to G4s in living cells. In order to detect the G4 interactome in living cells, Zhang *et al*. developed a strategy called co‐binding‐mediated protein profiling (CMPP)^[^ ^107^ ^]^ (Figure 5B). CMPP utilizes a cell‐permeable, functionalized G4‐ligand probe. This probe consists of three functional parts, which are a PyPDS group for recognizing the G4 structures, a photoreactive alphatic diazirine group, and the alkyne group for conjugating azide‐biotin. After treated with the probe, the cell culture was irradiated with 365 nm light to induce the probe to conjugate to the proximal G4BPs. Then the labeled G4BPs were enriched by streptavidin magnetic beads and identified by label‐free LC‐MS/MS. More than 200 G4BPs were detected with CMPP. Similar principle has been used to develop G4 ligand‐mediated cross‐linking and pull‐down (G4‐LIM‐CAP) to study G4BPs in living cells.^[^ ^111^ ^]^ Su *et al*. used the G4‐LIM‐CAP method to detect the G4BPs in SV589 and MM231 cells and discovered 8 new G4BPs. Both two works reveal that the type and abundance of G4BPs significantly vary in different types of cells. These methods have expanded our understanding of G4‐protein interactions in living cells. Some G4BPs may only express in a special physiological condition, which cannot be detected in normal cell culture conditions. Future works are expected to pay more attention in the profiling of G4BPs in different cellular contexts. However, the existing methods report the G4BPs from all the intracellular G4 sites, which cannot provide the relationship between G4BPs and the specific G4 containing genes. Analyzing the G4BP at a certain locus of G4s remains the major question of this field in the future.

## 7 BIOCHEMICAL TARGETING G4s IN LIVING CELLS

Since G4s are widely present in the genome and transcriptome of living cells and have diverse biological functions, it is very promising to explore biomedical applications based on G4s. G4s have been proved to mediate eIF4A‐dependent translation of proto‐oncogenes^[^ ^51^ ^]^ and considered as a new target for tumor therapy. There has been great progress towards the connection between G4 formation and synthetic lethality in cancer cells.^[^ ^8^, ^16^, ^17^ ^]^ Recently, Zhao *et al*. reported that SARS‐COV‐2 may be inhibited by the G4 structures in its genomic sequence,^[^ ^18^ ^]^ which further enhanced the attractiveness of therapeutic applications targeting G4s. The increasing understanding about the properties of G4s has promoted to develop a repertoire of molecular and chemical tools for the targeting of G4s.

### 7.1 Biochemical targeting G4s by small molecule ligands

G4 small molecule ligands can not only be used in fluorescent imaging of G4s in cells, but also can regulate the biofunction of G4s in living cells.^[^ ^26^ ^]^ In general, these ligands regulate the function of G4s mainly through two ways. Firstly, G4 ligands can enhance the stability of G4 structures and subsequently interfere their biofunctions. Secondly, the G4 ligands can disturb the interaction between the G4BPs and G4s. Based on this principle, G4 ligands have been verified to regulate a variety of biological functions, including translation,^[^ ^112^ ^]^ the telomere structure,^[^ ^113^ ^]^ proto‐oncogene expression,^[^ ^114^ ^]^ RNA splicing,^[^ ^54^ ^]^ local epigenetic state of chromatins,^[^ ^36^ ^]^ and so on (Table 4).

**TABLE 4:** Representative G‐quadruplex ligands for in vivo regulation

**G4 ligands**	**Applications**	**Regulatory effects**	**Ref**.
PDC12	Chicken DT40 cells	Histone H3K9 and DNA cytosine methylation	[36]
CX‐5461	Human HCT116 cells and patient‐derived xenografts (PDX) models	DNA damage and selective lethality	[118]
PDS	Human HT1080 cells	Telomere dysfunction and long‐term growth arrest	[115]
TMPyP4	Zaire ebolavirus	Inhibition of intracellular replication	[125]
EPI	In vitro	Conversion of telomeric G‐quadruplex structures	[113]
RGB‐1	Human HEK293 cells and MCF‐7 cells	Inhibition of translation	[112]
PDS and CarboxyPDS	Adult neural stem cell and progenitor cells derived from the adult mouse subventricular zone	Inhibition of proliferation	[126]
GQC‐05	Human HeLa cells	Alternative splicing	[54]
Cephaeline	Human HEK 293FT cells	Alternative splicing	[71]
ZnAPC	Human MCF‐7 cells	NRAS mRNA breaking	[114]
GTC365	Human MCF‐7 and UACC cells	Restoring tertiary DNA structures in mutant hTERT promoters	[116]
CM03	Human PDAC cells	Cell death	[117]
CX‐3543	Human NHA and GSC cells and xenograft models	Genomic instability	[119]
TMPyP4	Zebrafish embryos	Inhibition of transcription	[122]

It is worthy to detailly introduce the exploration of using G4 ligands in tumor therapy. It was firstly discovered about 10 years ago that PDS can inhibit the growth of tumor cells.^[^ ^115^ ^]^ In the last decade, synthetic lethality in cancer cells via G4 ligands has been examined extensively. A small drug‐like pharmacological chaperone (pharmacoperone) molecule, GTC365, can cause the hTERT promoter G4 to misfold and induce death of tumor cells.^[^ ^116^ ^]^ Pancreatic ductal adenocarcinoma (PDAC) has a variety of tumor‐related genes in which G4s are enriched in promoter regions. Targeting these genes through G4 ligands can produce anti‐tumor effects.^[^ ^117^ ^]^ CX‐5461 can block replication forks and induce DNA damage after stabilizing the DNA G4 structures. Patient‐derived xenograft model has shown that BRCA1/2‐deficient tumors were very sensitive to this G4 ligand.^[^ ^118^ ^]^ Similarly, CX‐3543 has a good therapeutic effect on ATRX‐deficient tumors.^[^ ^119^ ^]^ Currently, CX‐5461 has been put into clinical trials, which shows a great promising of G4 targeted therapeutics.

Small molecule ligands can regulate G4 biofunctions via many different pathways. These ligands bind to the G4 structures at multiple sites in the genome and transcriptome, which has been confirmed by cell imaging experiments.^[^ ^75^ ^]^ Therefore, the ligand for regulating transcription may also interfere translation, resulting in complex and unpredictable final regulatory results. How to specifically regulate the function of G4s is the key goal for this field. Recently, Dickerhoff *et al*. have reported a new drug‐like small molecule PEQ, which can bind MYC promoter G4s in a sequence‐specific way.^[^ ^120^ ^]^ This result provides the first example of single‐target regulation of G4s and will promote the rational design of small molecule ligands targeting individual G4s.

### 7.2 Targeting G4s with enhanced specificity

In addition to G4 ligands, there have been other kinds of G4 regulators, which have enhanced specificity. In other words, these G4 regulators can target G4 in a specific gene, giving a specific regulatory result. Antisense oligonucleotides (ASOs) are short‐stranded nucleic acids that can be designed to hybridize to specific sequences of DNA or RNA in living cells, thereby mediating RNA degradation or DNA transcription inhibition.^[^ ^121^ ^]^ ASO has been used to unwind the G4 structures on a specific promoter region and down‐regulate the expression of the corresponding gene.^[^ ^122^ ^]^ Besides, the modulation of G4s can also be precisely induced through the regulation of G4BPs. For example, the small molecule inhibitor of G4BP NME2 can inhibit the expression of the tumor gene hTERT.^[^ ^123^ ^]^ However, although this method targets a single protein, G4BPs such as NME2 can bind to multiple G4 sites throughout the genome, and its regulatory outcomes may still be diverse. Another strategy to target the G4 structure by protein is to bind the G4‐forming regions through the dCas9/sgRNA protein complex.^[^ ^124^ ^]^ dCas9 has a strong binding affinity to nucleic acids and can unwind the G4 structures, thus affecting G4‐dependent biological functions. This strategy is expected to become an effective tool to target and regulate gene‐specific G4 structures. However, dCas9 is a large exogenous protein, which is not easy to be introduced into living cells. Therefore, biochemical tools that target individual G4s are always under demand.

## 8 CONCLUSIONS AND PERSPECTIVES

In the last decade, the methodological advances in G4 detection and biochemical targeting have greatly expanded our understanding of the function and mechanism of intracellular G4s. There are three aspects of advances that are worth highlighting. Firstly, the recognition and targeting tools of G4s have been developed to be more powerful. The first small molecule ligand that can selectively bind to G4 structures at sequence‐specific level, PEQ,^[^ ^120^ ^]^ has been screened and tested in vivo. Moreover, a small peptide G4 probe^[^ ^103^ ^]^ has been reported to detect more chromatin G4s with a high signal‐noise ratio. Secondly, in situ imaging of G4 in the cells has reached monogenic and single‐cell level.^[^ ^86^ ^]^ mRNA G4s of a specific gene can be detected in many genes and many cell lines. Thirdly, the omics methods have been able to profile the G4 landscape in an intact physiological state, which can give more exact data of intracellular G4s. Although further improvement is needed, G4 ChIP‐seq^[^ ^34^ ^]^ and G4 CUT&Tag^[^ ^94^ ^]^ have achieved the profiling of G4 in cellular chromatin context. In addition, in situ recognition and labeling methods for characterizing G4BPs have been realized by CMPP^[^ ^107^ ^]^ and G4‐LIM‐CAP.^[^ ^111^ ^]^ Taking advantage of these imaging and omics methods, the formation, distribution, localization, dynamics, and interactions have been discovered, providing a systematic landscape of intracellular G4s.

Overall, these genome and transcriptome scale studies provide future directions to unravel the G4‐associated biological processes. There are still unanswered and arising questions in fundamental biology. Future research is expected to advance our understanding of the detailed mechanisms of the intracellular G4s and unravel the underlying biological consequences, and their connections to human diseases. However, the currently applicable methods remain in their infancy for solving these questions. Firstly, it remains possible that existing sequencing methods cannot detect the full entirety of intracellular G4s. Secondly, transient and dynamic G4 structurome and interactome in living cells may not be accurately detected with current technologies. Thirdly, there are only few tools that can be used to target and regulate intracellular G4s at single‐motif resolution. Lastly, the detection and regulation methods for G4s have rarely been tested with clinic samples for further development of diagnostic and therapeutic applications. The development of novel methodologies with superior resolution, throughput, and sensitivity will provide groundbreaking discoveries and applications that would be useful in the clinic in the future.

## CONFLICT OF INTEREST

The authors declare no competing financial interests.
